# Supplementary material for: 3D Electrospun Polycaprolactone Scaffolds to Assess Human Periodontal Ligament Cells Mechanobiological Behaviour
Source: Biomimetics (Basel). 2023 Mar 7;8(1):108. doi: 10.3390/biomimetics8010108 (PMC10046578; doi:10.3390/biomimetics8010108)
Supplement: Supplementary file 1 [file biomimetics-08-00108-s001.zip › biomimetics-2271654-supplementary.pdf]

Supplementary materials

Table S1: Absolute stiffness (in 10^{-3} N.µm^{-1}) values for all the samples at day 0 and day 21 for the three different series. The subscripts 1, 2 and 3 correspond to the 3 samples in each series. Samples from the same column (with the same name and subscript) are not associated. NS-Stat : Non-seeded static scaffolds.

Series	K (10^{-3} N/µm)	NS-Stat$_1$	NS-Stat$_2$	NS-Stat$_3$	Stat$_1$	Stat$_2$	Stat$_3$	Dyn$_1$	Dyn$_2$	Dyn$_3$
1st	d_0	9.1	9.0	7.7	5.7	6.8	7.9	6.6	14.3	9.2
	d_{21}	7.3	8.9	7.3	2.3	4.5	5.0	4.1	9.3	4.7
2nd	d_0	1.9	2.4	1.8	3.5	1.9	2.8	1.2	7.4	2.1
	d_{21}	1.5	1.6	2.0	1.0	1.2	2.3	0.9	3.1	2.0
3rd	d_0	5.3	3.9	6.2	6.8	7.3	10.6	7.9	7.9	6.8
	d_{21}	4.9	6.2	5.8	4.6	7.1	6.6	4.7	3.9	3.9

Table S2: Mean and standard deviation (SD) of the ALP activity measured on the triplicate for each condition and timestep.

ALP activity (U.10^{-5}/day)		Day 10		Day 21	
		Stat	Dyn	Stat	Dyn
1st series	Mean	0.31	3.77	1.71	1.89
	SD	0.16	2.68	0.59	0.66
2nd series	Mean	0.51	1.82	2.03	0.51
	SD	0.70	0.78	0.23	0.09
3rd series	Mean	0.59	0.53	0.51	0.86
	SD	0.03	0.01	0.01	0.01

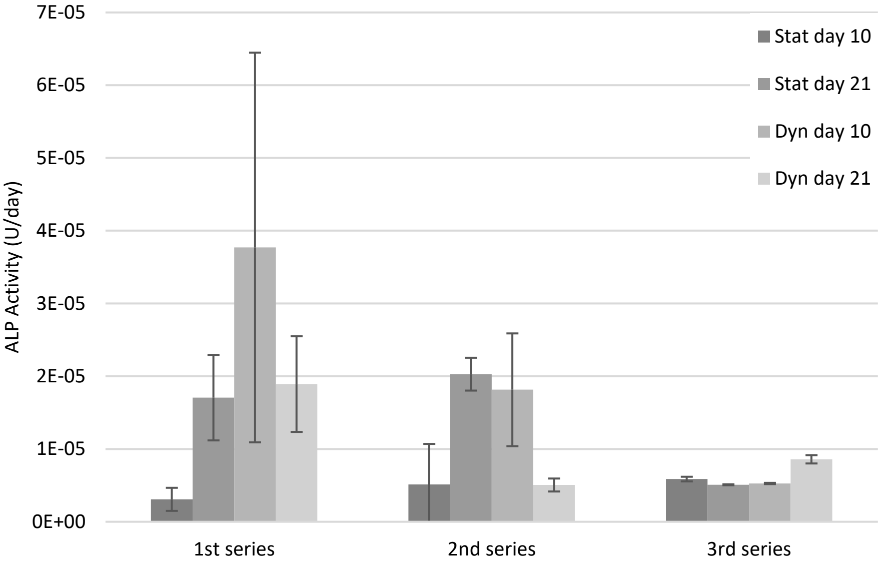

Figure S1: Bar charts of ALP activity averaged values and SD for the two conditions, the two time-steps, and the three experimental series

Table S3: Mean and standard deviation (SD) of the mass of IL-6 measured on the triplicate for each condition and timestep.

mass Il-6 (pg/day)		Day 10		Day 21	
		**Stat**	**Dyn**	**Stat**	**Dyn**
**1st series**	Mean	15.9	15.9	48.1	8.2
	SD	1.8	3.8	6.1	1.2
**2nd series**	Mean	9.7	12.4	9.9	2.3
	SD	1.8	3.5	1.4	0
**3rd series**	Mean	2.9	3.2	66.2	3.6
	SD	0.7	2.4	2.7	1.2

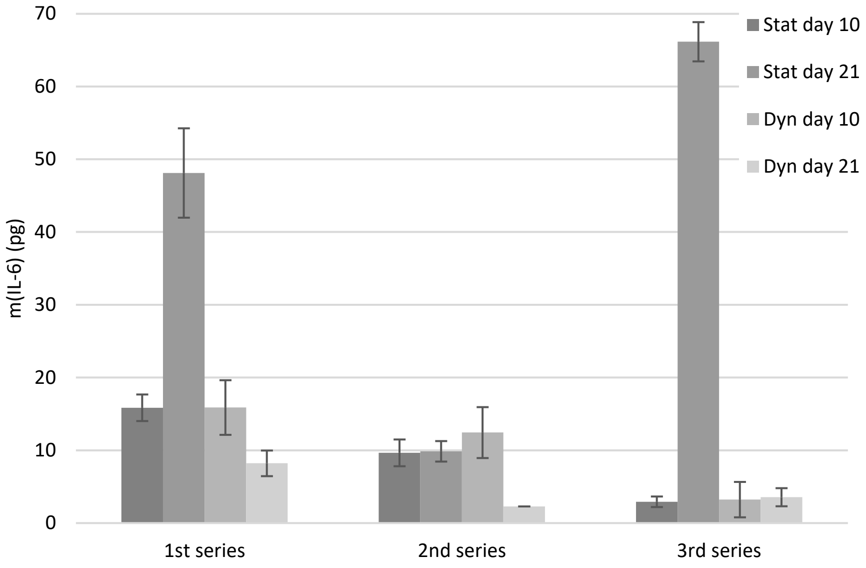

Figure S2: Bar charts of mass of IL-6 averaged values and SD for the two conditions, the two time-steps, and the three experimental series
